# Magnetism-mediated targeting hyperthermia-immunotherapy in “cold” tumor with CSF1R inhibitor

**DOI:** 10.7150/thno.57511

**Published:** 2021-05-03

**Authors:** Yuefei Fang, Yang He, Canhao Wu, Meng Zhang, Zeyun Gu, Jiaxin Zhang, Ergang Liu, Qin Xu, Akmal M. Asrorov, Yongzhuo Huang

**Affiliations:** 1 Artemisinin Research Center, Guangzhou University of Chinese Medicine, 12 Jichang Rd, Guangzhou 510450, China.; 2 State Key Laboratory of Drug Research, Shanghai Institute of Materia Medica, Chinese Academy of Sciences, 501 Haike Rd, Shanghai 201203, China.; 3 University of Chinese Academy of Sciences, Beijing, China.; 4 Zhongshan Institute for Drug Discovery, The Institutes of Drug Discovery and Development, CAS, Zhongshan 528437, China.; 5 NMPA Key Laboratory for Quality Research and Evaluation of Pharmaceutical Excipients, Shanghai 201203, China.

**Keywords:** magnetic hyperthermia, CSF1R inhibitor, tumor microenvironment, tumor-associated macrophage, immune memory, targeted delivery, magnetic liposomes

## Abstract

**Background:** Immunotherapy has profoundly changed the landscape of cancer management and represented the most significant breakthrough. Yet, it is a formidable challenge that the majority of cancers - the so-called “cold” tumors - poorly respond to immunotherapy. To find a general immunoregulatory modality that can be applied to a broad spectrum of cancers is an urgent need.

**Methods:** Magnetic hyperthermia (MHT) possesses promise in cancer therapy. We develop a safe and effective therapeutic strategy by using magnetism-mediated targeting MHT-immunotherapy in “cold” colon cancer. A magnetic liposomal system modified with cell-penetrating TAT peptide was developed for targeted delivery of a CSF1R inhibitor (BLZ945), which can block the CSF1-CSF1R pathway and reduce M2 macrophages. The targeted delivery strategy is characterized by its magnetic navigation and TAT-promoting intratumoral penetration.

**Results:** The liposomes (termed TAT-BLZmlips) can induce ICD and cause excessive CRT exposure on the cell surface, which transmits an “eat-me” signal to DCs to elicit immunity. The combination of MHT and BLZ945 can repolarize M2 macrophages in the tumor microenvironment to relieve immunosuppression, normalize the tumor blood vessels, and promote T-lymphocyte infiltration. The antitumor effector CD8^+^ T cells were increased after treatment.

**Conclusion:** This work demonstrated that TAT-BLZmlips with magnetic navigation and MHT can remodel tumor microenvironment and activate immune responses and memory, thus inhibiting tumor growth and recurrence.

## Introduction

Due to the high immunosuppression in the tumor microenvironment, there are a majority of solid tumors with a poor response rate to immunotherapy 1, 2. For example, only 5% of colorectal cancer patients have DNA mismatch repair deficient (dMMR) or microsatellite instability-high (MSI-H) 3, which can be classified into “hot” tumors that yield positive outcomes from immunotherapy. But the majority of patients with “cold” tumors do not benefit from immunotherapy. How to turn “cold” into “hot” by modulating the tumor microenvironment has become an important field in oncology 4.

Magnetic nanoparticle-mediated hyperthermia is an effective and safe method of cancer therapy. The magnetic nanoparticles in the tumor can generate heat under the external alternating magnetic field (AMF) 5. In general, the temperature ranging from around 40 °C to 45 °C can selectively kill the tumor cells but spare the normal cells 6. Unlike photothermal therapy that is typically applied in superficial tumors owing to the limited penetrating ability of beams in human tissues, magnetic hyperthermia (MHT) can work remotely in the deep parts of the body 7, 8. The anti-cancer effects of MHT, such as DNA damage, Fenton reaction, and mechanical shear force, can lead to tumor cell necrosis and apoptosis 9-11. Importantly, magnetic hyperthermia has a variety of effects on the tumor immune microenvironment 12-14. These treatment strategies not only directly act on the cancer cells but also carry out immune effects on dendritic cell (DC) activation and T cell infiltration and thus elicit anticancer immunity. The combination therapy of MHT and drugs has attracted much attention. For instance, MHT in combination with immune checkpoint inhibitors yields a synergistic effect on overcoming tumor metastasis and recurrence 15, 16. It thus provides a novel avenue for multimodal immunotherapy.

Macrophage colony-stimulating factor 1 (M-CSF1 or CSF1) plays an important role in immunosuppression 17. CSF1/CSF1R signaling is crucial for the differentiation of mononuclear cells, especially macrophages, and maintains the immunosuppressive and protumor functions of tumor-associated macrophages (TAMs) 18. TAMs are abundant in the tumor microenvironment and characterized by CSF1R overexpression, and promote tumor growth and metastasis, as well as suppress cytotoxic T lymphocytes 19, 20. Besides, CSF1R is also expressed in DCs, neutrophils, and myeloid-derived suppressor cells (MDSCs), and its expression is often associated with the diminished survival of cancer patients 21-23. Therefore, CSF1/CSF1R blockade by using CSF1R inhibitors is a promising anticancer immunotherapy strategy 24.

In this work, we propose a combination therapy strategy by co-applying a CSF1R inhibitor (BLZ945) and MHT to remodel the tumor immune microenvironment, yield the synergistic MHT-immunotherapy, and thus inhibit tumor growth and recurrence. We developed a magnetic liposome system modified by cell-penetrating peptide (CPP), for achieving magnetic targeting delivery and CPP-mediated intratumor penetration. It was expected that low-power MHT under AMF induced immune responses that synergistically work with a CSF1R inhibitor BLZ945.

## Results and Discussion

### Preparation and Characterization of TAT-BLZmlips

**Figure 1:**
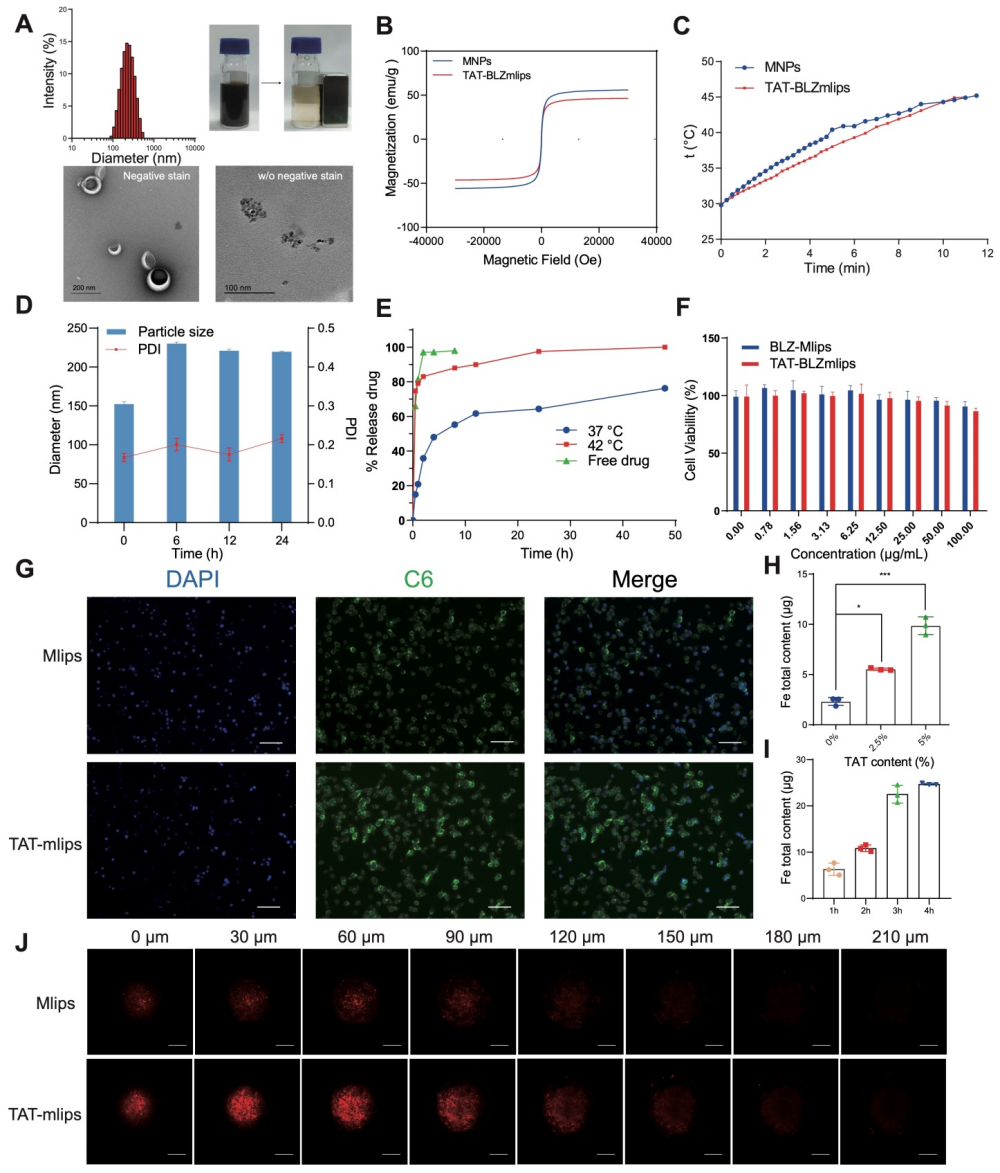
Characterization of TAT-BLZmlips. (A) Particle size, magnetic adsorption, and TEM image of TAT-BLZmlips. (B) Magnetic hysteresis loop of TAT-BLZmlips at 300 K. (C) Heating efficiency of TAT-BLZmlips. (D) Particle size measured in PBS containing 10% FBS. (E) Drug release curves of TAT-BLZmlips at different temperatures. (F) The viability of tumor cells after treatment with TAT-BLZmlips; it shows little cytotoxicity of the magnetic materials and BLZ945. (G) Fluorescent images of cell uptake, scale bar: 100 µm. (H) Cellular uptake of TAT-BLZmlips modified with different TAT densities in CT26 cells. (I) The cellular uptake efficiency at various incubation times. (J) Fluorescent images of penetration of tumor spheroids, scale bar: 200 µm.

The magnetic liposomes (termed TAT-BLZmlips) modified with the cell-penetrating TAT peptide and containing superparamagnetic nanoparticles (MNPs) and BLZ945 were prepared, with an average particle size of 149 ± 1.6 nm and positive ζ-potential (Supporting information, Figure S1A-D). The MNPs showed the size of a crystal particle was less than 10 nm. Transmission electron microscopy (TEM) images revealed an obvious core-shell structure of TAT-BLZmlips, indicated the MNPs were encapsulated in liposomes. Meanwhile, the magnetic liposomes with magnetism were readily adsorbed by a magnet (Figure 1A). The encapsulation efficiency was 80.1% for BLZ945 and 64.8% for MNPs, and the loading capacity was 4.8% for BLZ945 and 1.8% for Fe. The saturation magnetization of the MNPs and TAT-BLZmlips were measured to be 55.9 and 46.2 emu/g, and they exhibited a similar pattern in both remnant magnetizations and coercivities (Figure 1B). As a result, their heating efficiency was similar as shown by the heating curve (Figure 1C). The heating rate was enhanced along with the elevating concentration of TAT-BLZmlips, revealing the dose-effect dependency. When the iron concentration was 4 mM, the temperature increased by 8 °C within 10-min exposure to AMF (Figure S1F). In the stability test, the magnetic liposomes showed good storage stability (Figure S1E). The size of TAT-BLZmlips was slightly increased to 220 nm when exposed to a serum-containing medium due to the surface adsorption of serum proteins, but remained stable afterward (Figure 1D). The drug release was temperature-dependent due to the heat-induced phase transition of DPPC; 80% of the drug quickly released within 1 h at 42 °C but there was slow release at 37 °C (Figure 1E).

The TAT-BLZmlips (up to 100 μg/mL) showed little cytotoxicity to the tumor cells without MHT treatment (i.e., no AMF exposure) (Figure 1F), suggesting the biocompatibility of this nano-system. In addition, BLZ945 is a CSF-1R inhibitor that targets macrophages but does not directly act on the cancer cells, and thus showed little cytotoxicity to the CT26 cells. The lipid-TAT was confirmed by 1H NMR and ESI-MS (Figure S1G-H). Due to surface modification of TAT, the uptake efficiency of TAT-mlips in the tumor cells was 4 folds higher than the control Mlips (without TAT) (Figure 1G-H), and reached a plateau of accumulation at 4 h (Figure 1I). The experiment of penetration of tumor spheroids showed that TAT-mlips had a stronger tumor permeability and reached the tumor interior (Figure 1J).

### *In vitro* study of TAT-BLZmlips-mediated MHT

**Figure 2:**
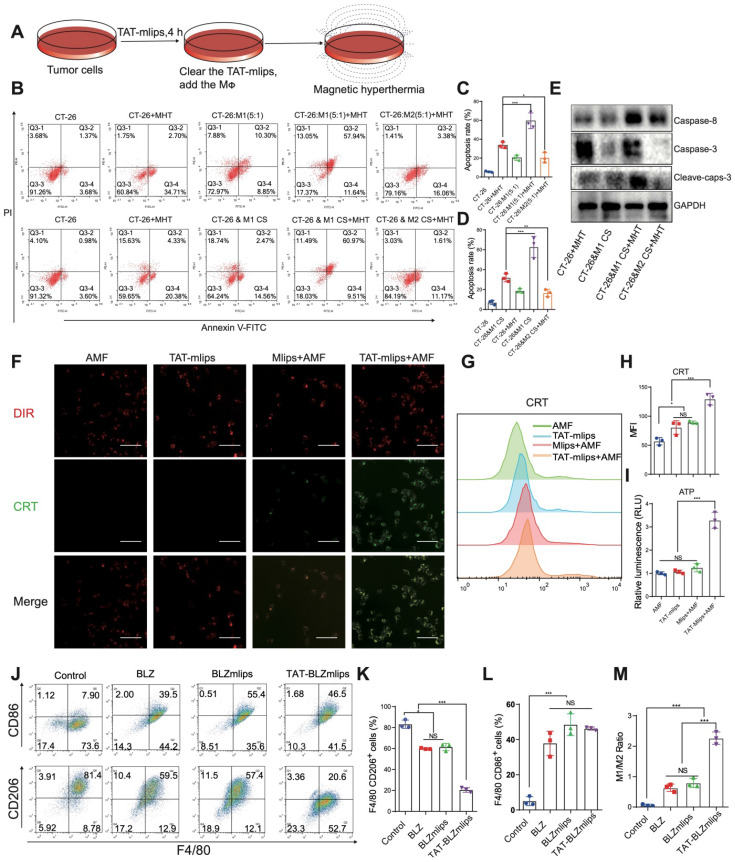
TAT-BLZmlips mediated magnetic hyperthermia *in vitro*. (A) Scheme of the treatment procedure. (B) Flow cytometry scattergram of tumor cell apoptosis. (C) Statistical analysis of apoptosis rate in MΦ/CT26 coculture. (D) Statistical analysis of the apoptosis rate in a non-direct cell-cell contact study. (E) Western blotting assay of the tumor cells after TAT-mlips-mediated MHT. (F) Fluorescent images of colocalization of CRT and cell membrane, scale bar: 100 µm. (G, H) Quantification of CRT exposure in CT-26 cancer cells after treatment. (I) Quantity analysis of the released ATP. (J) The dot plots of CD86^+^ or CD206^+^ MΦ. Statistical analysis of the F4/80^+^/CD206^+^ MΦ (K), F4/80^+^/CD86^+^ MΦ subsets (L), and the ratio of M1/M2Φ (M) after treatments.

By mimicking the tumor microenvironment, a MΦ/CT26 cell co-culture model was used in the MHT-induced apoptosis study (Figure 2A). The results showed that M1Φ significantly increased the apoptotic rate (58%) of the CT26 tumor cells that were treated with MHT induced by TAT-mlips and AMF, compared to 25% in the M2Φ/CT26 group under the same condition (Figure 2B-C). Furthermore, in a non-direct cell-cell contact condition, the apoptotic rate of the tumor cells treated with MHT induced by TAT-mlips and AMF was also remarkably enhanced when exposed to the M1Φ culture supernatant (Figure 2D). According to the western blotting assay, the apoptosis signaling pathways of the tumor cells was activated by M1Φ (Figure 2E). It demonstrated that M1Φ can enhance MHT-induced apoptosis in the tumor cells.

### TAT-BLZmlips-mediated MHT for ICD induction

Immunogenic cell death (ICD) can result in the antigen release from the dead tumor cells, thus promoting DC maturation and activating T cell-based immunity 25. Treatment with TAT-mlips and AMF significantly induced ICD in the CT26 tumor cells, evidenced by a large amount of CRT on the cell membrane (Figure 2F) and a higher level of the released ATP than other treatment groups (Figure 2I). The results were further confirmed by flow cytometry (Figure 2G-H). Importantly, the tumor cells with the ICD effect efficiently stimulated the DC maturation that are characterized by high co-expression of surface CD86/CD80 molecules (Figure S2A-B), when the ICD-induced tumor cells were given and co-cultured with the DCs.

### The effects of TAT-BLZmlips on the immune cells

The effect of TAT-BLZmlips on MΦ polarization was investigated. The expression of CD86 (M1 marker)/CD206 (M2 marker) was used to indicate the M1/M2 ratio. The TAT-BLZmlips treatment (w/o MHT) significantly enhanced the M1/M2 ratio to 2.06 due to the enhanced uptake mediated by TAT, compared to 0.63 and 0.72 of BLZ945 and BLZmlips, respectively (Figure 2J-M). It demonstrated that the CSF-1R inhibitor BLZ945 delivered by TAT-BLZmlips had efficient M2-to-M1 repolarization. Our result was consistent with other reports that CSF-1R inhibition can cause MΦ repolarization 24, 26. Furthermore, the effect of MHT mediated by TAT-mlips (without BLZ945) on the immune cells was also explored. The TAT-mlips-based MHT treatment was able to repolarize the MΦ from M2 to M1 phenotype, reflected by the decreased CD206 and increased CD86 (Figure S2C-F). Reactive oxygen species (ROS) is a driving factor for M1-phenotype differentiation. ROS can activate mitogen-activated protein kinase (MAPK) and NF-κB pathways, and consequently produce inflammatory factors and promote M1 polarization 27. MHT initiated the increase of ROS in the cells and thus could induce the repolarization of M2 to M1 (Figure S2G). Moreover, our study also revealed that the TAT-mlips-based MHT promoted DC maturation (Figure S2H-I). It suggested that MHT is a promising tool for reprogramming the immune cells (e.g., macrophages and DCs) toward the anticancer phenotypes, as well as inducing the ICD effect in the cancer cells, thus regulating the tumor immune microenvironment.

### The pharmacokinetics and biodistribution of TAT-BLZmlips in rats

The pharmacokinetics profiles of BLZ945 and TAT-BLZmlips were shown in Figure S3A. The blood concentration decrease of BLZ945 in the TAT-BLZmlips group was slower than that of free drugs after administration. The circulation half-life of TAT-BLZmlips was 5.5 h, much longer than the BLZ945 group (1.2 h). The area under the curve (AUC) of TAT-BLZmlips (56.2 μg/mL⋅h) was about 2.6 times greater than that of BLZ945 (21.2 μg/mL⋅h). The biodistribution of free BLZ945 and TAT-BLZmlips (w/o AMF) is illustrated in Figure S3B. It should be noted that there was an increased drug distribution to the liver in the group of TAT-BLZmlips and further study needs to be performed to explain the phenomenon.

### Magnetic navigation increased the intratumoral accumulation of TAT-BLZmlips

**Figure 3:**
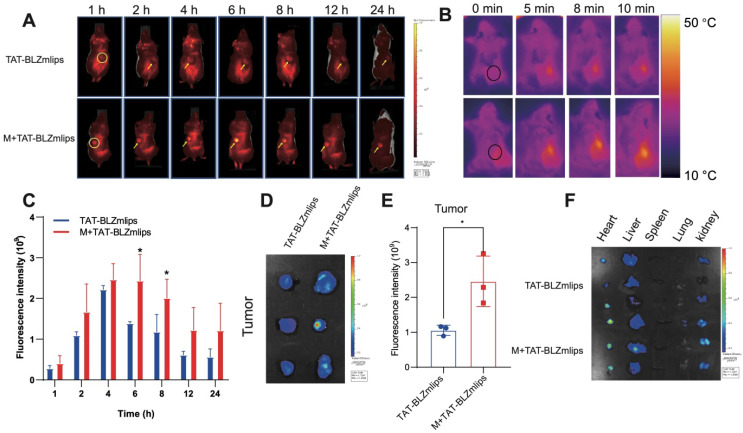
Magnetic navigation increased the intratumoral accumulation of TAT-BLZmlips. (A) The *in vivo* imaging photos. (B) The Infrared imaging of MHT. (C) Statistical results of *in vivo* imaging at the tumor sites. (D) *Ex vivo* imaging of the tumors. (E) Statistical results of *ex vivo* imaging at the dissected tumors. (F) *Ex vivo* imaging of the major organs. Three mice per group (n = 3).

The bio-distribution of the Cy5-labeled TAT-BLZmlips was investigated in the CT26 tumor-bearing mice. The fluorescence spectra of the liposomes were characterized (Figure S3C). The *in vivo* imaging results showed that TAT-BLZlimps had the highest accumulation at 6 h post-injection under magnetic navigation (Figure 3A, C, Figure S3D). The quantitative analysis in the dissected tumor showed that the accumulation of the TAT-BLZmlips with magnetic navigation (termed M+TAT-BLZmlips) was significantly higher than the TAT-BLZmlips without magnetic navigation (Figure 3D-E). Accordingly, the temperature at the tumor during the MHT after magnetic navigation (about 42 °C) was significantly higher than that in the non-targeted group (Figure 3B). But in the normal organs, there was no significant difference between the two groups (Figure 3F, Figure S3E).

### TAT-BLZmlips-mediated mild MHT eradicated the CT26 tumors

**Figure 4:**
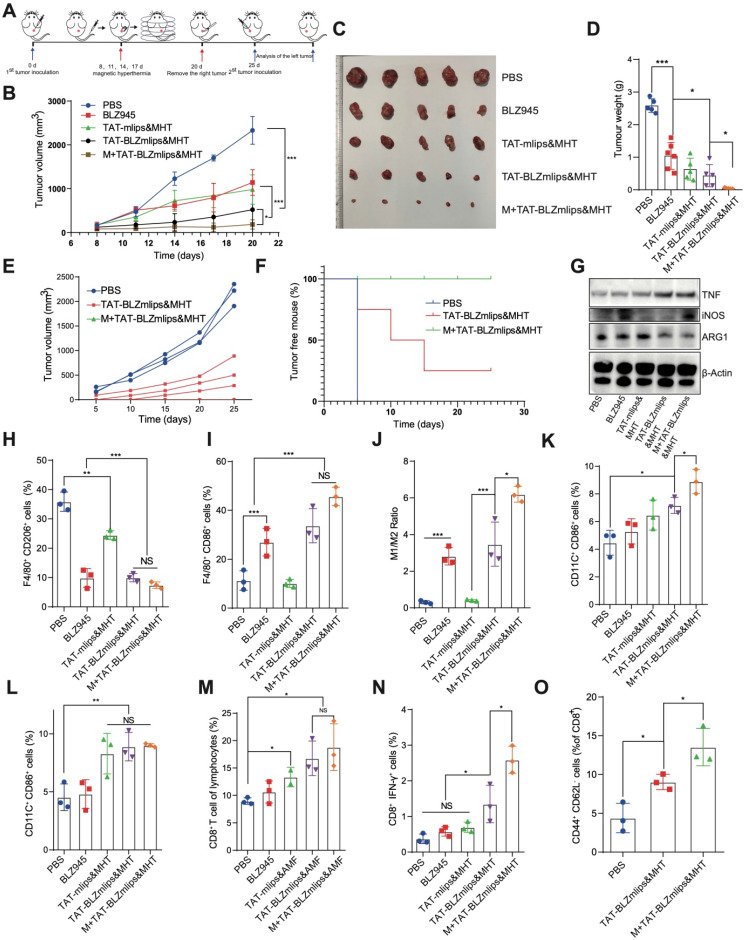
TAT-BLZmlips mediated mild MHT and CSF1R inhibition in cancer therapy. (A) Schematic illustration of the therapy regimen. (B) The tumor volume curves. Photographs (C) and tumor weight (D) at the end of the first regimen. (E) The tumor volume curve after inoculating the re-challenged tumor cells (n=3 for PBS group, and n =5 for other two groups). (F) Percentage of tumor-free animals (G) Western blotting assay of iNOS, TNF-α, and ARG1 in the tumors after treatment. (H) F4/80^+^/CD206^+^ M2Φ subsets in the tumors after treatment. (I) F4/80^+^/CD86^+^ M1Φ subsets in the tumors after treatment. (J) The ratio of M1/ M2Φ in the tumors after treatment. (K) CD11C^+^/CD86^+^ DC subsets in the lymph nodes after treatment. (L) CD11C^+^/CD86^+^ DC subsets in the tumors after treatment. (M) CD8^+^ T cells subsets in the tumors after treatment. (N) CD8^+^/IFN-γ^+^ T cells subsets in the tumors after treatment. (O) Typical flow cytometry plots of effector memory T cells (CD8^+^ CD44^+^ CD62L^-^) in the spleen after tumor re-inoculation. Five mice per group (n = 5).

The TAT-BLZmlips were characterized by three advantages: 1) magnetic navigation for targeting delivery; 2) MHT effect; and 3) codelivery of a CSF-1R inhibitor for immunotherapy. CT26 colon cancer is characterized by microsatellite stability and the consequent less-immunogenic nature 28, 29, which is often used as a “cold” tumor model 30. The treatment regimen in the mice bearing CT26 tumor is shown in Figure 4A. The TAT-BLZmlips with magnetic navigation and MHT (termed M+TAT-BLZmlips&MHT) significantly arrested tumor growth, with the highest efficacy among all groups (Figure 4B-D), demonstrating the synergistic effect of thermal-immunotherapy and the benefit of magnetic targeting drug delivery. It was interesting that there was no statistical difference between BLZ945 and TAT-mlips&MHT in the tumor growth curve, which revealed that the mild MHT and CSF-1R inhibition yielded a similar treatment outcome in this tumor model. The inhibition rate of tumor growth was 55.4% for BLZ945, 75.5% for TAT-mlips&MHT, 83.1% for TAT-BLZmlips&MHT, 98.2% for M+TAT-BLZmlips&MHT, respectively.

To further evaluate the immune memory against tumor recurrence, the tumors in the treated mice above were surgically removed at the end of the first regimen. After the surgical wound was healed, the tumor cells were transplanted on another hind-thigh side as a challenge. As shown in Figure 4E, the contralateral tumors grew rapidly in the control PBS group. By contrast, in the group treated with TAT-BLZmlips&MHT (without magnetic navigation) in the first regimen, there were two mice with the complete annihilation of the re-challenged tumor cells, while three showed the recurred tumor but with slow growth (Figure 4F). Of note, in the group pretreated with M+TAT-BLZmlips&MHT (with magnetic navigation), there was no recurred tumor found in all animals, exhibiting the strong anticancer immune memory that efficiently eradicated the re-challenged tumor cells. The results showed that the TAT-BLZmlips with co-application of BLZ945 and MHT not only effectively inhibited the tumor growth but also activated immune memory to prevent tumor recurrence.

Finally, we evaluated the preliminary safety of the TAT-BLZmlips. There was no hemolysis in an *in vitro* test (Figure S4F). The bodyweight monitoring and histopathological examination indicated that there was no obvious side toxicity (Figure S4E, S5).

### The mechanism study of TAT-BLZmlips-based thermal immunotherapy

At the experiment endpoint, the dissected tumors were used to detect the immune responses. Figure 4G shows the increased iNOS and TNF-α, but the decreased ARG1 in the M+TAT-BLZmlips&MHT group. TNF-α is a main anti-tumor cytokine from M1Φ; iNOS is an M1-related marker while ARG1 is an M2-related marker. The result indicated the M2-to-M1 repolarization of TAM. The flow cytometry results showed a decreased amount of intratumoral M2Φ but an increased amount of M1Φ in the M+TAT-BLZmlips&MHT group, further confirming the switch from M2 to M1 (Figure 4H-J, S4A). There were an increased amount of the mature DCs in the tumors and draining lymph nodes near the tumors in the M+TAT-BLZmlips&MHT group (Figure 4K-L, S4B). It demonstrated that this treatment activated the MΦ and DCs in the tumors. As a result, the CD8^+^IFN-γ^+^ T cell population in the M+TAT-BLZmlips&MHT group was significantly higher than other groups (Figure 4M-N, S4C). The increase in the antitumor immune cells demonstrated the remodeling tumor immune microenvironment and activation of anticancer immunity.

To evaluate the long-term immunity, the amount of effector memory T cells in the spleen was measured. The results showed there were a significantly increased amount of effect memory T cells in the M+TAT-BLZmlips&MHT group (Figure 4O, S4D). The long-term memory effects in the peripheral immune organs are helpful for preventing tumor recurrence 31.

### The normalization of tumor blood vessels after treatment

**Figure 5:**
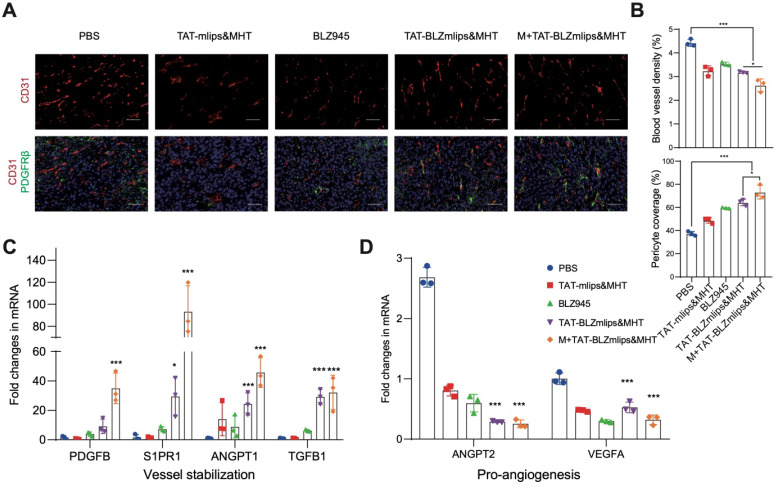
Remodeling tumor blood vessels. (A) Immunofluorescence staining of CD31 (red, tumor vessels) and PDGFRb (green, pericytes), scale bar: 200 μm. (B) Quantitative analysis of CD31^+^ blood vessel density and PDGFRb^+^ pericytes. (C) Expression of vessel-stabilizing factors in the tumors. (D) Expression of pro-angiogenesis factors in the tumors.

TAMs play an important role as a pro-angiogenic factor in the formation of abnormal tumor blood vessels 32. Our previous report revealed that the M2-to-M1 repolarization of TAM promoted the normalization of neovascularization via a mechanism of reducing TGF-β and HIF-1α, thus suppressing the TGF-β/VEGF/VEGFR2/HIF-1α axis against angiogenesis 33. In addition, mild hyperthermia can increase blood perfusion and oxygen supply, which also promotes blood vessel normalization 34. The abnormal structure of tumor vessels, characterized by the high density of vessels and poor pericyte coverage, leads to various malignant outcomes in the tumor microenvironment, such as tumor metastasis and immunosuppression 35, 36. Tumor vessel normalization can enhance the delivery of drugs and the infiltration of lymphocytes to improve the treatment efficacy 37, 38. Therefore, tumor vasculature normalization is a useful therapeutic avenue 39. The phenotype of macrophages in the tumor changed after treatment, then we detected the morphology of tumor blood vessels. According to immunofluorescence staining, the blood vessel density of M+TAT-BLZmlips&MHT in the tumors decreased and the pericyte coverage was improved, reflected by the reduced CD31 (a vessel marker) and increased platelet-derived growth factor receptor b (PDGFRb, a pericyte marker), compared to the PBS group (Figure 5A-B). In addition, the qPCR results showed that the pro-angiogenic genes (ANGPT2 and VEGFA) were downregulated whereas the vessel maturation-related genes (PDGFB, S1PR1, ANGPT1, and TGFB1) were significantly up-regulated (Figure 5C-D). It indicated that the treatment with M+TAT-BLZmlips&MHT promoted tumor blood vessel normalization. The TAT-BLZmlips with MHT and CSF1R inhibition effectively normalized the tumor blood vessels, which could play an important role in remodeling the tumor immune microenvironment.

## Conclusion

This work developed a safe and effective magnetic liposome system for delivering a CSF-1R inhibitor and conducting MHT. This liposomal system can enhance tumor-targeted delivery by magnetic navigation and cell-penetrating peptide modification. It was demonstrated that the proposed MHT-immunotherapy yielded a potent treatment efficacy in the colon cancer model and it was also able to activate long-term immune memory to prevent tumor recurrence. This combination therapy strategy lifted the immunosuppression by repolarizing TAMs, activating DCs, promoting the infiltration of cytotoxic T lymphocytes, and normalizing the tumor blood vessels. The TAT-BLZmlips provide a promising drug delivery and treatment strategy for MHT-immunotherapy.

## Experimental section

### Materials

BLZ945 and NHS-Cy5 were obtained from Meilun Biotechnology (Dalian, China). DPPC and DSPE-PEG2000-MAL were purchased from Avito Pharmaceutical Technology (Shanghai, China). Cell-penetrating peptide TAT (sequence: CYGRKKRRQRRR) was synthesized by Bankpeptide Biological Technology (Hefei, China), Dulbecco's modified Eagle's Medium (DMEM) cell culture medium, and fetal bovine serum (FBS) were from Gibco (Thermo Fisher Scientific, Waltham, USA). Annexin V-FITC Apoptosis Detection Kit and Enhanced ATP Assay Kit were purchased from Beyotime (Shanghai, China). The 3-(4, 5-dimethyl-2-thiazolyl)-2, 5-diphenyltetrazolium bromide (MTT), cocktail protease inhibitors, and lipopolysaccharide (LPS) were obtained from Sigma-Aldrich (St. Louis, USA). Recombinant murine macrophage colony-stimulating factor (M-CSF), interferon-gamma (IFN-γ), and interleukin-4 (IL-4) were purchased from Peprotech (Cranbury, USA). The qPCR SYBR® Green Master Mix, cDNA synthesis SuperMix kit, and TRIeasy™ Total RNA Extraction Reagent were from Yeasen Biotech (Shanghai, China). All primers for qPCR were synthesized by Generay Biotech (Shanghai, China). DiR (1,1-dioctadecyl-3,3,3,3-tetramethylindotricarbocyaine iodide) was purchased from AmyJet Scientific (Wuhan, China). All flow cytometric antibodies (CD8-PE, IFN-γ-PE-Cy7, CD11C-FITC, CD86-APC, CD206-PE, and F4/80-FITC) for flow cytometry purchased from BD Biosciences (Franklin Lakes, USA).

### Cell lines

CT-26 colorectal cancer cells were obtained from Shanghai Cell Bank of Chinese Academy of Sciences (Shanghai, China). The cells were cultured in 1640 medium with 10% FBS, 100 U/mL of streptomycin and 100 U/mL of penicillin at 37 °C in a humidified incubator with 5% CO_2_.

### Animals

Balb/c nude mice (female, 3-4 weeks) and Balb/c mice (6-8 weeks) were provided from Shanghai Laboratory Animal Center (SLAC) (Shanghai, China) and housed at the SPF care facility under a 12 h light/dark cycle. All the animal procedures were approved by the Institutional Animal Care and Use Committee (IACUC) (permit number: SYXK (Shanghai) 2015-0027), Shanghai Institute of Materia Medica, Chinese Academy of Sciences.

### Preparation and characterization of the TAT-BLZmlips

The superparamagnetic nanoparticles were synthesized by a method of co-precipitation using ferric chloride as raw material. FeCl_3_ (507 mg) and Na_2_SO_3_ (128 mg) were dissolved in 50 ml deionized water. The solution was stirred in a 55 °C water bath for 15 min, and then 2.5 ml ammonia water was added to the solution rapidly for 30-min reaction, and 2 ml sodium citrate solution (0.6 g/mL) was subsequently added for continuous reaction at 80 °C for 30 min. The thus-formed magnetic nanoparticles were separated by magnets and thoroughly washed until the pH of the supernatant was neutral. The characterization was conducted by a dynamic light scattering instrument (Nano-ZS90, Malvern, UK) and transmission electron microscope (Talos L120C, FEI, USA).

DSPE-PEG_2000_-MAL and TAT (molar ratio 1:1.2) were dissolved in PBS and reacted at room temperature under the protection of N_2_ for 24 h. After dialysis, the product DSPE-PEG_2000_-TAT was determined by 1H NMR and ESI-MS.

DPPC, cholesterol, and DSPE-PEG_2000_-TAT at a molar ratio of 69:26:5 were dissolved in a mixture solvent (chloroform and methanol 1:1), then mixed with BLZ945 chloroform solution. The organic solvents were removed by rotary evaporation to form a uniform film. The aqueous dispersion (2 ml) containing 10 mg magnetic nanoparticles was added to hydrate the film. The liposome suspension was subjected to probe ultrasound for 5 min and then purified by Sephadex™ G-50 (GE Healthcare, USA) column. Cy5 and Coumarin 6 labeled magnetic liposomes were prepared according to the above method.

The characterization of TAT-BLZmlips was conducted by a dynamic light scattering instrument (Nano ZS 90, Malvern, UK) and a transmission electron microscope (Talos L120C TEM, Thermo Fisher). The BLZ945 encapsulation efficiency and drug-loading capacity of the NPs were determined by HPLC (1260 Infinity, Agilent technologies, USA). The HPLC conditions: the mobile phase was a mixture of Solvent A (H_2_O + 0.1% TFA) and Solvent B (Acetonitrile + 0.1% TFA) at a flow rate of 1.0 mL/min using a C18 column (250 × 4.6 mm, Agilent, USA) gradient elution in the room temperature (25 °C). The detection wavelength was 280 nm. The gradient elution was set as table 1.

The stability of the NPs was evaluated in the PBS (pH 7.4) containing 10% newborn bovine serum by monitoring the size change at the different time points (up to 14 days). By using a dialysis membrane (MWCO 10-12 kDa) method, the in-vitro drug release of the NPs was evaluated in PBS (pH 7.4) adding 0.5% (w/v) SDS with 150 rpm/min shaking at 37 °C or 42 °C. The released BLZ945 was determined by HPLC as described above.

### The measurement of heating efficiency

In order to test their heating efficiency, the NPs and TAT-BLZmlips with the same Fe content (10 mM) were dispersed in water, and then exposed to an alternating magnetic field (288 kHz, 44 mT) for heating to the same temperature (measured by optical fiber probe). The heating time was recorded.

### Cytotoxicity assay, cellular uptake, and apoptosis analysis *in vitro*

The CT26 cells were seeded into the 96-well plates at a density of 5 × 10^3^ cells/well overnight. The cells were treated with the BLZmlips or TAT-BLZmlips at different concentrations for 24 h. Cell viability was detected by a standard MTT method.

The CT26 cells were seeded in the 12-well plates with 5 × 10^4^ per well overnight. The cells were treated with TAT-BLZmlips or C6-labeled TAT-BLZmlips (50 μg/mL, Fe content) for 4 h. The cells were washed twice with PBS and then observed by fluorescence microscope. Subsequently, the cells were collected for lysis. The cell lysate was centrifuged at 12,000 rpm for 15 min, and the precipitated magnetic nanoparticles were dissolved by adding 1 ml HCl (5 mol/L). The total intracellular Fe was measured by a standard colorimetry method 40.

The CT-26 cells were seeded into the 12-well plates at a density of 1×10^5^ cells overnight. The TAT-mlips were added to each well at a concentration of Fe (10 μg/mL) for 4-h culture with the tumor cells. The cells were then washed with PBS to remove the free TAT-mlips and cultured in a fresh medium. The bone marrow-derived M2 or M1 macrophages were added and co-cultured with the CT26 cells at a ratio of 1:5. They were then exposed to an alternating magnetic field (288 kHz, 44 mT) for 10 min. After 24 h, the apoptosis rate was detected by flow cytometry.

Because the lately-added macrophages did not contain TAT-mlips and there was no MHT effect generated in the macrophages. Therefore, the apoptotic portion was thus contributed solely by the tumor cells with MHT.

### *In vitro* calreticulin and ATP assay

The CT26 cells were seeded in a cell culture dish (35 mm) at a density of 1 × 10^4^ per dish overnight. The cells were treated with the TAT-Mlips (20 μg/mL) for 4 h and then thoroughly washed. The cells in a fresh medium were exposed to an alternating magnetic field (AMF) (288 kHz, 44 mT) for 10 min. After 6 h, the cells were collected and stained with Alexa fluor 488 anti-CRT antibody and DiR. The CRT expression was detected by flow cytometry and immunofluorescence staining. The ATP level in the cell supernatant was determined by ATP Assay Kit according to the manufacture protocol.

### Culture and polarization of mouse bone marrow-derived macrophages (BMDM)

The BMDM were cultured and induced polarization using a general procedure. In brief, the bone marrow cells were collected from the Balb/c mice femur by rinsing them out with serum-free DMEM medium. The cells were cultured in fresh DMEM medium containing 30 ng/mL M-CSF for 72 hours to induce differentiation. Afterward, IFN-γ (20 ng/mL) and LPS (100 ng/mL) were added to the medium for 24 h to induce M1-phenotype polarization, or IL-4 (40 ng/mL) to induce M2 phenotype polarization.

### *In vitro* macrophage polarization and DC stimulation

The TAT-mlips (20 μg/mL) were added to the bone marrow-derived macrophages or DCs (1×10^5^ cells) for 4 h. After wash, the cells in a fresh culture medium were exposed to AMF (288 kHz, 44 mT) for 10 min, and after 24 h, the cells were then collected for detection of F4/80^+^CD206^+^CD86^+^ macrophage and CD11C^+^/CD86^+^ DCs.

### The pharmacokinetics and biodistribution of TAT-BLZmlips

The SD rats were intravenously injected with BLZ945 and TAT-BLZmlips (BLZ945 dosage: 5 mg/kg). The blood samples were collected by retro-orbital bleeding after anesthesia, and the serum was obtained, and then the triple volume of acetonitrile was added to the serum. After centrifugation, BLZ945 in the supernatant was detected by HPLC. At the endpoint, the rats were euthanasia to collect heart, liver, spleen, lung, and kidney. The organs were homogenized in 0.9% NaCl solution, and then added the triple volume of acetonitrile. After centrifugation, BLZ945 in the supernatant was determined by HPLC as described above.

### *In vivo* imaging study

The subcutaneous xenograft colorectal tumor model was established by inoculated with CT26 cells (3 × 10^5^ cells per mouse) in the back of Balb/c nude mice. The mice with tumors (300-400 mm^3^) were divided into two groups randomly (with or without magnetic navigation) and intravenously injected with an equal dose of Cy5-labeled TAT-BLZmlips. In the magnetic navigation group, a small round magnet with a diameter of 8 mm was placed on the tumor site for magnetic navigation. The mice were exposed to the IVIS imaging system (Caliper PerkinElmer, USA) at the pre-set time to monitor the biodistribution of the liposomes. At the endpoint (36 h post-injection), the mice were euthanized and the major organs and tumors were collected for *ex vivo* imaging.

### Antitumor and anti-recurrence treatment

The Balb/c mice were inoculated with CT26 cells (3 × 10^5^ cells per mouse) in the hind thigh. When the tumor volume was about 60 mm^3^, the mice were randomly divided into 5 groups (PBS, BLZ945, TAT-mlips&MHT, TAT-BLZmlips&MHT, and M+TAT-BLZmlips&MHT). The mice were intravenously injected at a dose of BLZ945 (5 mg/kg) every three days for four times. Right after injection, a small round magnet with a diameter of 8 mm was placed on the tumor site for 6 h for magnetic navigation. The mice were exposed to an AMF (288 kHz, 44 mT) for 10 min. The tumor volume was calculated by the equation as follows.

Tumor volume = (width^2^ × length)/2

The tumor growth inhibition rate was calculated by the equation as follows:

[1 - (mean tumor weight of a treated group) / (mean tumor weight of PBS group)] × 100%

The first endpoint was determined by the tumor volume (~2000 mm^3^). Part of the mice was euthanized and the tumors and tissues were collected for further analysis. For other mice, the tumors were surgically removed from the mice (n = 5 for each group; there were two died in the PBS group after surgery, and thus three mice left in this group). After the wound healing, the mice were re-inoculated with CT-26 cells (2 × 10^5^ cells per mouse) on the contralateral side, and the volume of the tumors was recorded, When the tumor volume reached 2000 mm^3^, the spleen was separated for flow cytometry after the mice euthanized.

### Flow cytometry analysis

The tumor tissues were digested for one hour at 37 °C with collagenase and hyaluronidase (1 mg/mL) to prepare the single-cell suspension. The lymph nodes and spleen were added with PBS (1 mg/mL) and ground to prepare a single-cell suspension. The cells were incubated with PBS with 0.2% BSA on ice for 30 min, then washed with PBS. The cells were stained using CD8-PE, IFN-γ-PE-Cy7, CD11C-FITC, CD86-APC, CD206-PE, F4/80-FITC, CD44-FITC, or CD62L-APC (BD Biosciences, Franklin Lakes, USA) for flow cytometry assay according to a standard procedure.

### Statistical analysis

All data were analyzed by GraphPad Prism 8.0.1 software and were expressed as mean ± SD (n ≥ 3). Statistical analysis was conducted via two-tailed paired and unpaired student's t-tests to determine differences, respectively. P-value < 0.05 was considered statistically significant (*0.01 < P < 0.05; **0.001 < P < 0.01; ***P < 0.001).

## Supplementary Material

Supplementary figures and tables.

## Figures and Tables

**Table 1:** The gradient elution

Time (min)	A	B
0	80%	20%
2	80%	20%
7	5%	95%
10	0%	100%
12	0%	100%
12	80%	20%

## Abbreviations

AMF: alternating magnetic field
ANGPT-1: angiopoietin-1
ANGPT-2: angiopoietin-2
AUC: area under curve
BMDM: bone marrows derived macrophages
CPP: cell-penetrating peptide
CRT: calreticulin
CSF-1R: colony-stimulating factor 1 receptor
CTL: cytotoxic T lymphocyte
DC: dendritic cells
EGF: epidermal growth factor
HIF-1α: hypoxia-inducible factor-1α
ICD: immunogenic cell death
IFN-γ: interferon-γ
IGF: insulin-like growth factor
MDSCs: myeloid-derived suppressor cell
MHT: magnetic hyperthermia
MNPs: magnetic nanoparticles
MΦ: macrophage
NPs: nanoparticles
PDGF: platelet-derived growth factor
PD-1: programmed death receptor 1
PD-L1: programmed death-ligand 1
PI3K: phosphatidylinositol-3-hydroxy kinase
q-PCR: real-time quantitative polymerase chain reaction
ROS: reactive oxygen species
S1PR1: sphingosine-1-phosphate receptor 1
TAMs: tumor-associated macrophages
TAT: transcriptional activator protein
TFA: trifluoroacetic acid
TGF-β: transforming growth factor-β
TIME: tumor immune microenvironment
TNF: tumor necrosis factor
VEGF: vascular endothelial growth factor
