# Supplementary material for: Magnetism-mediated targeting hyperthermia-immunotherapy in “cold” tumor with CSF1R inhibitor
Source: Theranostics. 2021 May 3;11(14):6860–72. doi: 10.7150/thno.57511 (PMC8171105; doi:10.7150/thno.57511)

Supporting information

Magnetism-Mediated Targeting Hyperthermia-Immunotherapy in "Cold" Tumor with CSF1R Inhibitor

Yuefei Fang [1,2,#], Yang He [2,3,#], Canhao Wu [1,2], Meng Zhang [2], Zeyun Gu [2,3], Jiaxin Zhang [2], Ergang Liu [4], Qin Xu [1,], Akmal M. Asrorov [2], Yongzhuo Huang [2,3,4,5,*]*

Table S1 Primers sequences of qPCR

Name	Sequences
S1PR1	For 5' CAGCAAATCGGACAATTCCT 3'
	Rev 5' GCCAGCGACCAAGTAAAGAG 3'
ANGPT1	For 5' GATGTCAATGGGGGAGGTT 3'
	Rev 5' CTCTGACTGGTAATGGCAAAAATA 3'
PDGFB	For 5' GGCCGAGTTGGACCTGAACATGA 3'
	Rev 5' GAAGTTGGCGTTGGTGCGGTCTA 3'
ANGPT2	For 5' AATAAGCAGCATCAGCCAAC 3'
	Rev 5' TCAAGTTGGAAGGACCACAT 3'
EGF	For 5' CTTGTCATGCTGCTCCTCCTG 3'
	Rev 5' TGCGACTCCTCACATCTCTGC 3'
TGFB1	For 5' CCCAGCATCTGCAAAGCTC 3'
	Rev 5' GTCAATGTACAGCTGCCGCA 3'
VEGFA	For 5' TCTTCAAGCCATCCTGTGTG 3'
	Rev 5' ATCCGCATAATCTGCATGGT 3'
GAPDH	For: 5' CTGCCACCCAGAAGACTGTG 3'
	Rev: 5' GGTCCTCAGTGTAGCCCAAG 3'

Figure S1 (A) DLS of MNPs. (B) TEM of MNPs. (C) The original TEM image of **Fig 1A**. (D) Zeta potentials of the NPs. (E) The stability of TAT-BLZmlips in 14 days. (F) The heating efficiency of TAT-BLZmlips with different concentrations. (G) 1H NMR spectrum of TAT, DSPE-PEG2000-Mal, DSPE-PEG2000-TAT. (H) ESI-MS of DSPE-PEG2000-MAL and DSPE-PEG2000-TAT.

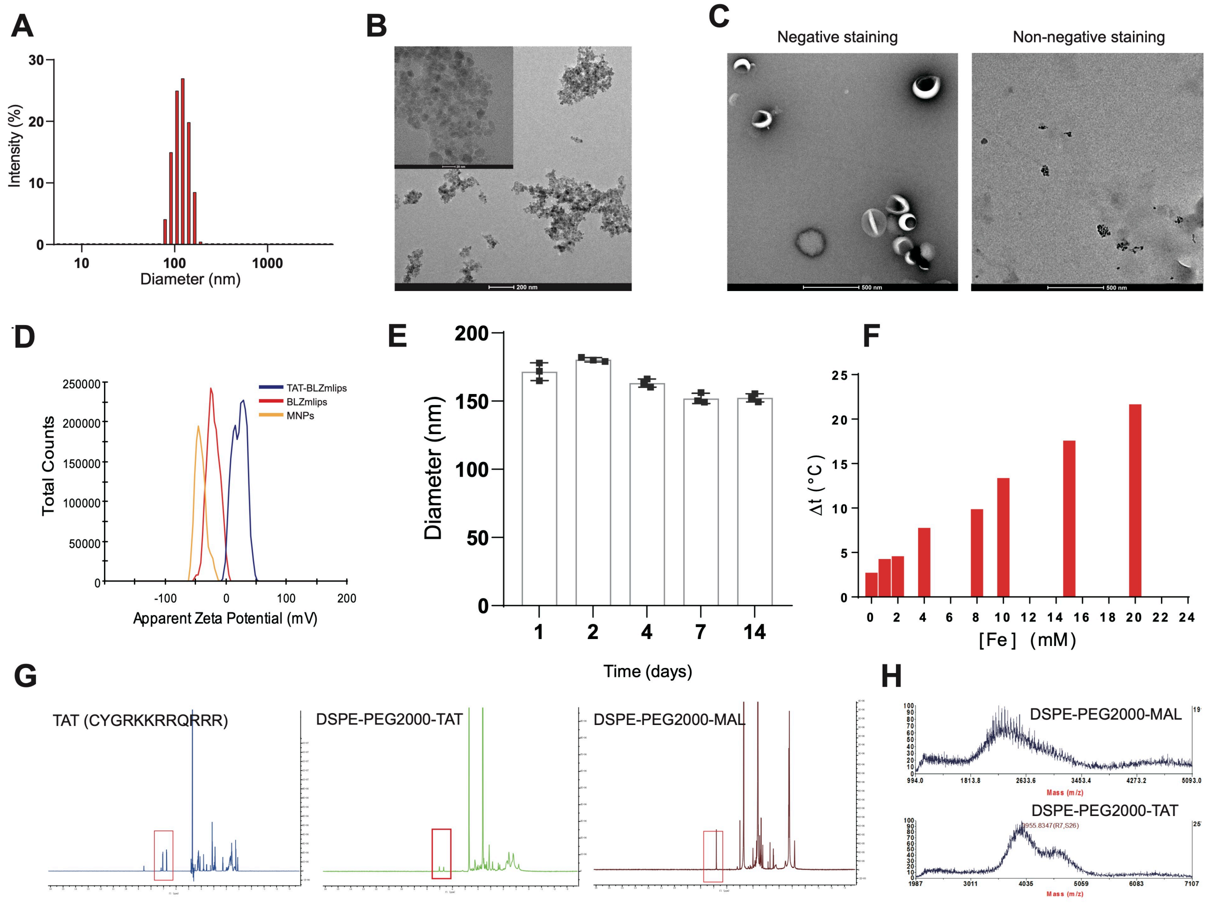

Figure S2 (A) Representative flow cytometric plots of the mature DC stimulated by the ICD-induced tumor cells. (B) The ICD effect of the cancer cells promoted the differentiation of $CD80^+/CD86^+$ DC subsets. (C) Representative flow cytometric plots of MΦ after treatment by TAT-mlips plus AMF. Statistical analysis of the $F4/80^+/CD206^+$ M2Φ (D), $F4/80^+/CD86^+$ M1Φ (E) subsets, and the ratio of M1Φ/M2Φ (F) after treatment by TAT-mlips plus AMF. (G) Intracellular level of ROS, scale bar: 50 μm. (H-I) Representative flow cytometric plots of the mature DC after treatment by TAT-mlips plus AMF.

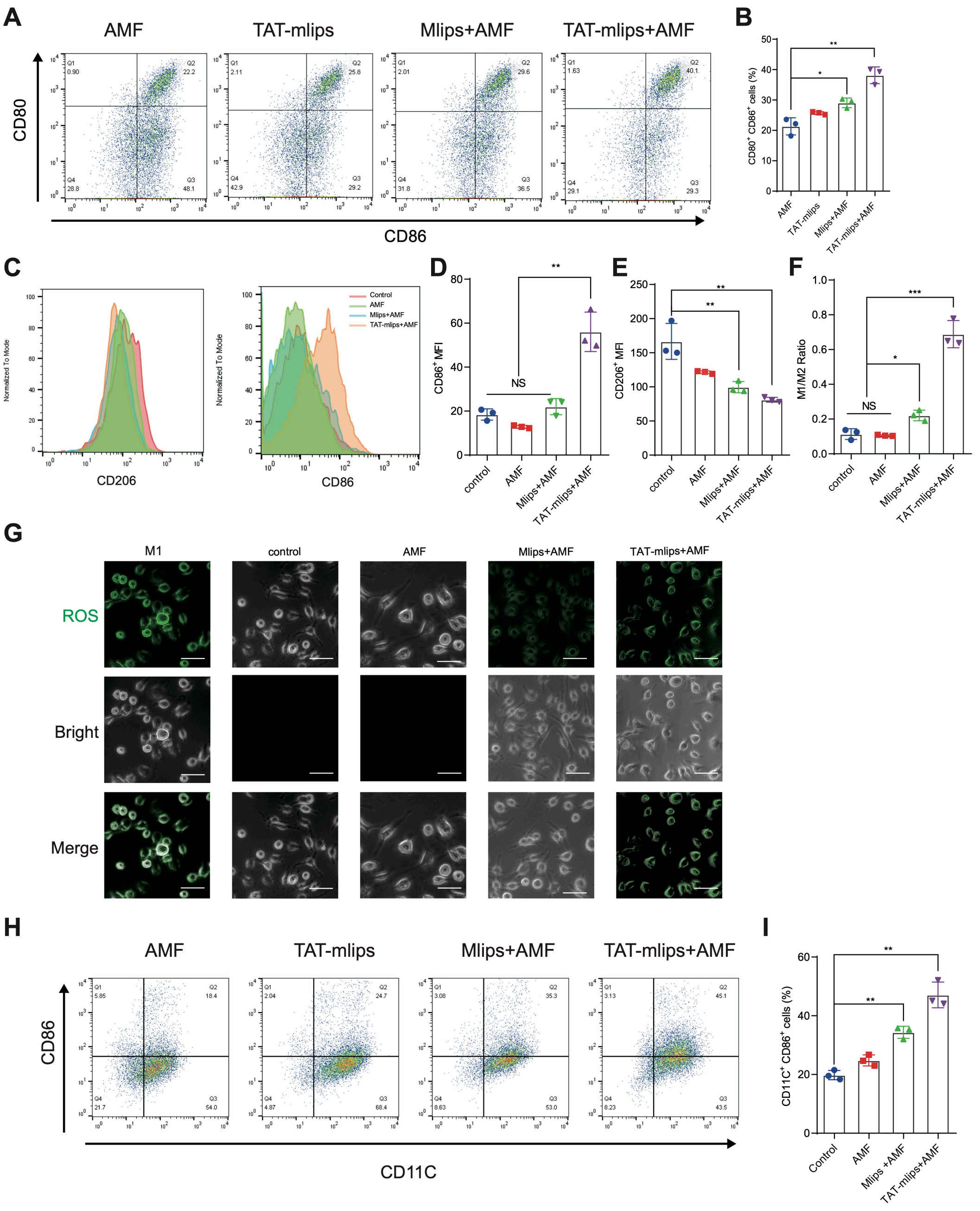

Figure S3 (A) The concentration of BLZ945 in plasma after intravenous administration of BLZ945 and TAT-BLZmlips (with the same BLZ945 dosage of 5 mg/kg). (B) Biodistribution of BLZ945 after intravenous administration of BLZ945 and TAT-BLZmlips. (C) The fluorescence spectra of Cy5, TAT-Cy5mlips and Cy5mlips. (D) Statistical results of the ROI of the tumor to normal tissue. (E) Statistical results of the in vivo imaging of the organs.

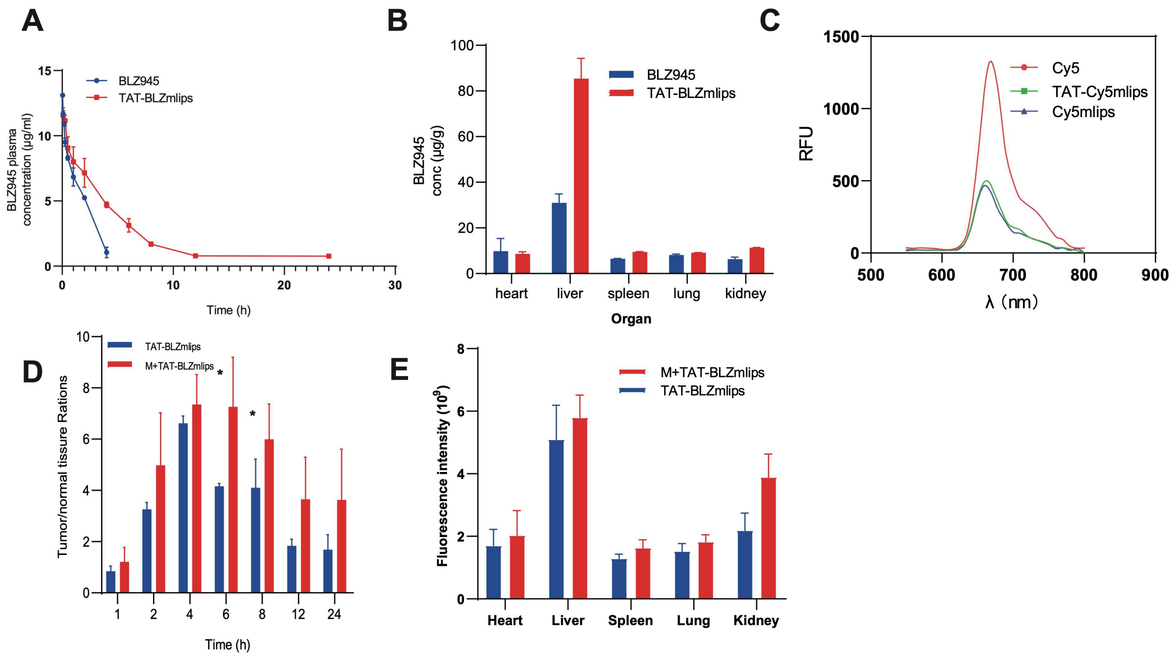

Figure S4 (A) Representative flow cytometric plots of F4/80$^+$/CD206$^+$ M2Φ and F4/80$^+$/CD86$^+$ M1Φ in the tumors. (B) Representative flow cytometric plots of CD11C$^+$/CD86$^+$ DC subsets in tumors and lymphonodus. (C) Representative flow cytometric plots of CD8$^+$/IFN-γ$^+$ T cells in the tumors. (D) Representative flow cytometric plots of the effector memory T cells (CD8$^+$CD44$^+$CD62L$^-$) in the spleens. (E) Bodyweight changes of the mice during treatment. (F) The in vitro hemolysis tests.

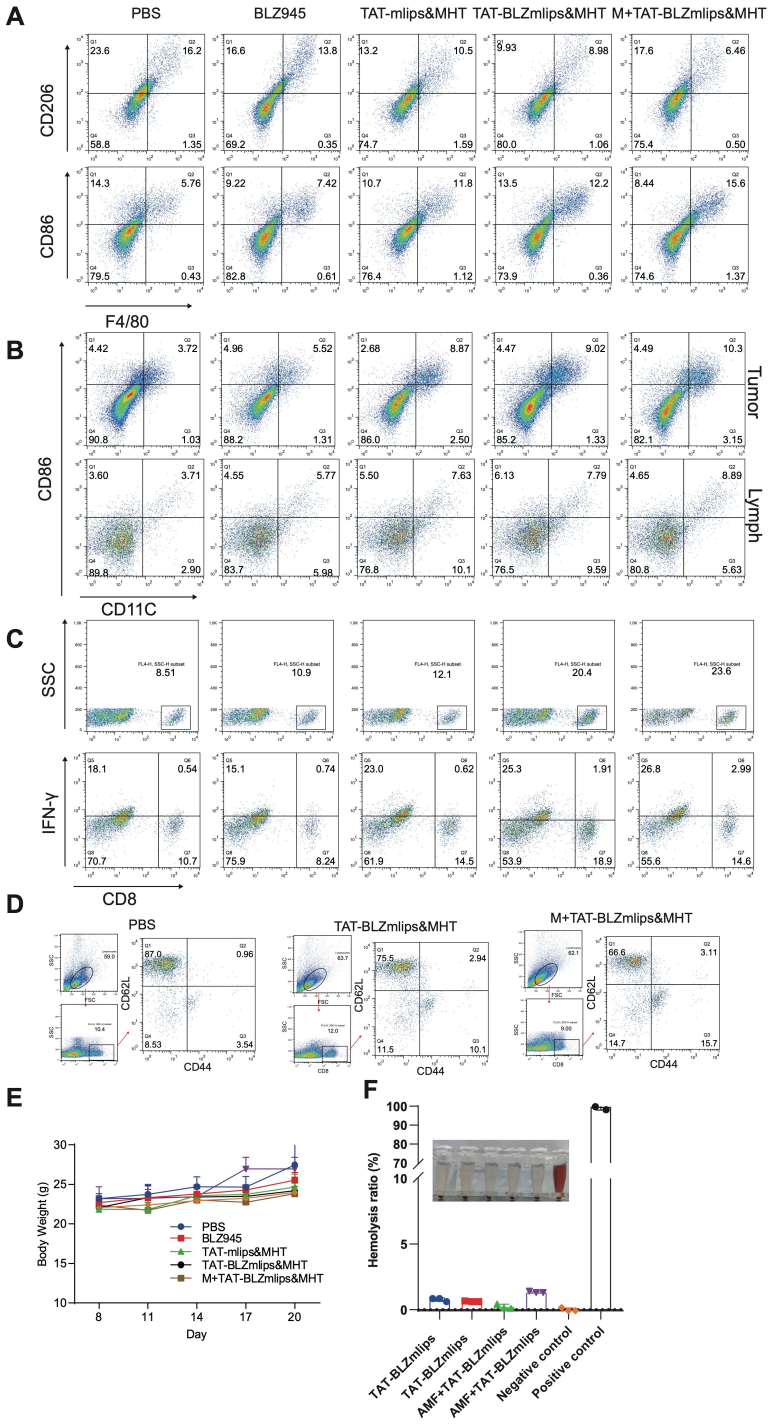

Figure S5 Histopathological examination of the major organs collected at the experimental endpoint (scale bar: 100 μm). No obvious lesions were found.

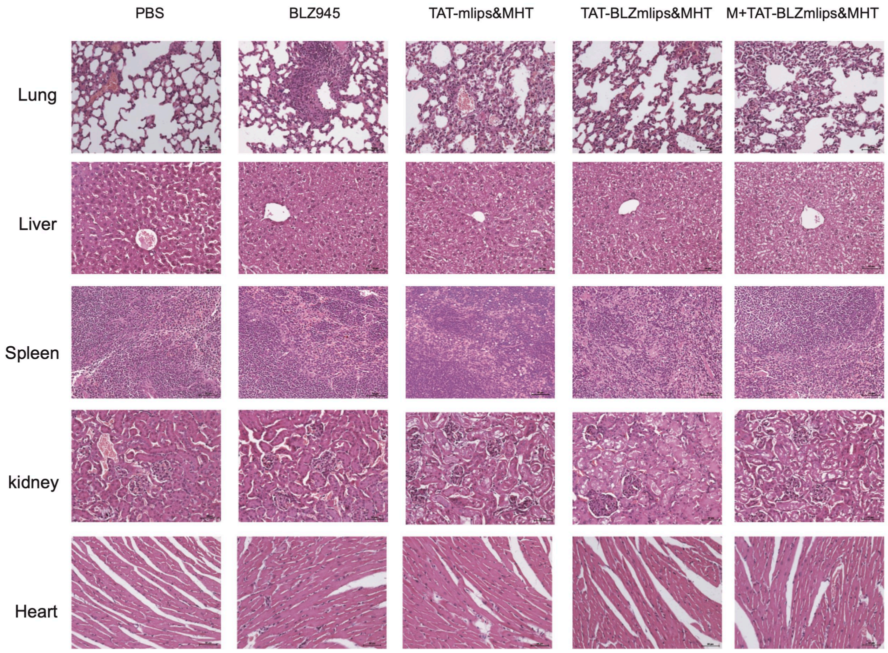

Supplement: Supplementary file 1 — Supplementary figures and tables. [file thnov11p6860s1.pdf]
